# Response of the Primary Auditory and Non-Auditory Cortices to Acoustic Stimulation: A Manganese-Enhanced MRI Study

**DOI:** 10.1371/journal.pone.0090427

**Published:** 2014-03-11

**Authors:** Hyungjun Kim, Junghun Cho, Young R. Kim, Youngkyu Song, Song-I Chun, Ji-Yeon Suh, Jeong Kon Kim, Yeon-Hee Ryu, Sun-Mi Choi, Hyungjoon Cho, Gyunggoo Cho

**Affiliations:** 1 Division of MR, Korea Basic Science Institute, Ochang-eup, Chungbuk, South Korea; 2 Division of Medical Research, Korea Institute of Oriental Medicine, Yuseong-gu, Daejeon, South Korea; 3 School of Nano-Bioscience and Chemical Engineering, UNIST (Ulsan National Institute of Science and Technology), Ulsan, South Korea; 4 Athinoula A. Martinos Center for Biomedical Imaging, Department of Radiology, Massachusetts General Hospital, Harvard Medical School, Charlestown, Massachusetts, United States of America; 5 Department of Radiology, University of Ulsan, Asan Medical Center, Songpa-gu, Seoul, South Korea; University of Salamanca- Institute for Neuroscience of Castille and Leon and Medical School, Spain

## Abstract

Structural and functional features of various cerebral cortices have been extensively explored in neuroscience research. We used manganese-enhanced MRI, a non-invasive method for examining stimulus-dependent activity in the whole brain, to investigate the activity in the layers of primary cortices and sensory, such as auditory and olfactory, pathways under acoustic stimulation. Male Sprague-Dawley rats, either with or without exposure to auditory stimulation, were scanned before and 24–29 hour after systemic MnCl_2_ injection. Cortex linearization and layer-dependent signal extraction were subsequently performed for detecting layer-specific cortical activity. We found stimulus-dependent activity in the deep layers of the primary auditory cortex and the auditory pathways. The primary sensory and visual cortices also showed the enhanced activity, whereas the olfactory pathways did not. Further, we performed correlation analysis of the signal intensity ratios among different layers of each cortex, and compared the strength of correlations between with and without the auditory stimulation. In the primary auditory cortex, the correlation strength between left and right hemisphere showed a slight but not significant increase with the acoustic simulation, whereas, in the primary sensory and visual cortex, the correlation coefficients were significantly smaller. These results suggest the possibility that even though the primary auditory, sensory, and visual cortices showed enhanced activity to the auditory stimulation, these cortices had different associations for auditory processing in the brain network.

## Introduction

Hearing is important for communication between individuals of the same species and for recognizing predators. Sound is processed via auditory pathways, which includes the cochlear nucleus (CN), inferior colliculus (IC), medial geniculate body (MGB), and primary auditory cortex (Aud) [1], [2]. It has been known that Aud, the neural crux of hearing, identifies the basic elements of sound, such as pitch and loudness. In addition, recent evidence suggests that primary sensory cortex (Sens) and primary visual cortex (Vis) are associated with the Aud [3], [4] and deeply related to the process of auditory information [5]–[8].

The network in rodent auditory system was explored using neuroanatomical tracing and immunohistochemistry [1], [2], [9]–[11]. Blood oxygen level-dependent (BOLD) MRI has been used for non-invasive investigations of the whole brain, which successfully delves into the rodent auditory system [12], [13]. However, BOLD MRI studies on the auditory system are difficult to interpret due to two reasons; the first is that there are challenges of distinguishing neural responses to a defined auditory stimulus from responses to background noise generated by the MRI scanner, and the second is that the auditory experiment is performed under anesthesia.

Manganese-enhanced MRI (MEMRI) detects activity-sensitive neural signals in awake, free-moving animals outside the MR scanner [14]–[16]. Manganese ions can pass through the blood-brain barrier (BBB), enter active neurons via voltage-gated calcium channels, and can be transported to adjacent neurons [17]. As a result, MEMRI identifies activity-dependent neural signals based on the accumulation of paramagnetic manganese ions [18]–[20]. To date, MEMRI has been used to delineate auditory pathways in free-moving rodents [14]–[16], [20]–[23], which demonstrates the ability of MEMRI to identify the brain network recruited during auditory processing.

However, despite the importance of Aud and association of Aud with Sens and Vis in processing auditory information, there are only a few reports regarding the comprehensive response of these cortices to auditory stimulation [3], [5]–[7]. In the present study, we used MEMRI, with aid of cortex linearization and layer analysis, to investigate cortical responses of Aud, Sens, and Vis layers to acoustic stimulation in free-moving rats.

## Materials and Methods

### 2.1. Animal preparation

All animal experimental procedures were approved by the Animal Care and Use Committee of the Korea Basic Science Institute (KBSI–0909). Thirty-two male Sprague-Dawley rats aged 9–10 weeks and with bodyweights of 300–350 g were used. Animals were maintained under normal conditions with an ambient temperature of 22–24°C, a 12 h light/12 h dark cycle, and with free access to food and water. Rats were pair-housed in single Plexiglas cages to reduce the stress induced by social isolation [24]. However, a highly aggressive partner is also a potential stress factor. Thus, the pair was excluded from the sample if 1 animal in the pair had a superficial wound [25]. For MEMRI, rats were injected intraperitoneally with 100 mM MnCl_2_ in saline, at a dose of 50 mg MnCl_2_/kg of body weight. Brain images were obtained twice: before and 24–29 h after the injection.

### 2.2. Auditory stimulation

After the manganese injection, 16 rats were placed in the silent room, and the other 16 were exposed to the auditory stimulation for 24 h. The pure-tone sound stimuli were in the frequency range of 10–20 kHz, and the amplitudes were modulated between 80 and 95 dB. The duration of the sound was 3–8 s, and sometimes the silent period was interrupted with a 10% probability to avoid habituation. For example, the sound stimulation could be presented in the following sequence: 1) 12 kHz, 85 dB, 3 s; 2) 18 kHz, 90 dB, 8 s; 3) silence 4 s; 4) 16 kHz, 95 dB, 7 s. These random sequences of sounds were generated using a in-house script written in Matlab (Mathworks, Natick, MA, USA) and were delivered through an audio speaker. The frequency and amplitude of the acoustic stimulus was measured and monitored during the experiments.

### 2.3. MRI acquisition

Images were acquired using a 4.7 T/40-cm horizontal magnet equipped with a Bruker BioSpec console, an actively decoupled quadrature rat head receive-only surface coil, and the transmit-only 72-mm volume RF coil. The surface coil was positioned using the rat's eye position to minimize inter-subject positional differences. T_1_-weighted images were acquired using a modified driven equilibrium Fourier transform (MDEFT) sequence with TE = 3.8 ms, TR = 15 ms, inversion delay = 1100 ms, flip angle = 15°, FOV = 3.2×3.2×2.58 cm^3^, and voxel size = 125×125×300 µm^3^. During the MRI scan, a warm water circulation system was used to maintain the animal's body temperature at 37°C, and <1.25% isoflurane in a 7∶3 mixture of N_2_O and O_2_ was delivered through the facemask. High-resolution T_1_-weighted images were obtained before and 1 day after intraperitoneal infusion of MnCl_2_ solution. On the scan, a thin tube containing 0.05 mM MnCl_2_ solution was placed on the surface coil, which was used for the external standard for the signal calibration.

### 2.4. Data processing

All images were aligned to the average brain template using affine and non-linear registration using FSL [26], [27]. For the comparison of the regional manganese uptake between the rats with and without the auditory stimulation, the average signal intensity was measured from several regions of interest (ROIs) including auditory, sensory, and visual cortices and auditory pathways, which process was repeated for olfactory pathways as negative control. Subsequently, the average signal intensities were normalized to adjacent temporalis muscle (TM) of each ROIs. This TM normalization compensates for both signal intensity gradients and inter-individual difference in MnCl_2_ uptake [21]. The ROIs of the primary auditory were manually segmented on the coronal slices from Bregma (Br) −3.0 to −6.8. For accurate delineation, segmentation of the Aud was conducted based on reference points located 1 mm apart (Figure S1). Primary sensory, visual cortices, and piriform (Pir)/lateral olfactory tract (LOT) were also depicted in the same way based on the rat brain atlas [28]. For the segmentation of ROIs of auditory pathways, cylinder-shaped ROIs were properly used as the region boundaries were not well-defined in the T_1_-weighted images, and a rectangular-shaped ROI was used for olfactory bulb (OB). On the delineation of TM ROIs, we used relatively superficial TMs for each ROI's normalization (Figure S2) because those parts include relatively small number of muscle types and the distribution of muscle fiber types is homogeneous [29]. After the normalization of ROIs by adjacent TMs, the signal intensity ratio (SIR) for each ROI was calculated based on the signal intensity (SI) of the pre- and post-injection images, which represented the cumulative manganese absorption caused by brain activity over 1 day:
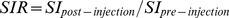
Where,
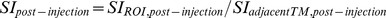


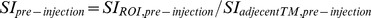



### 2.5. Cortical linearization

To explore the layer-specific response of the auditory cortex, we flattened the auditory cortex and extracted the mean SIR of each layer [30]. First, the ROI of the Aud was delineated according to the rat brain atlas [28]. As the rat cerebral cortex has a saddle-like curved surface, the cortex was linearized to construct a flat cortical map. The outline of the cortex was delineated and fitted to a fourth-order polynomial (Figure S1B). A line was drawn perpendicular to the polynomial every 125 µm along the fitted polynomial [30]. Subsequently, the fitted polynomial was linearized, and the lines perpendicular to the polynomial were placed parallel to each other (Figure S1C). On the perpendicular line, we sampled the SIRs every 25 µm [30]. In the flattened map of Aud, each layer was defined as a function of the depth from the outer fitted line. Layer I was defined between 0 µm to 200 µm, layer II/III was between 200 µm to 625 µm, layer IV was between 625 µm to 775 µm, layer V was between 775 µm to 1175 µm, and layer VI was between 1175 µm to 1500 µm (Figure S1D). The depth of each layer was decided based on the percentage depth of each Aud layer in rats with similar body weights that were used in a previous cytoarchitectural study [31]. This result was confirmed by the average signal intensity profile from MEMRI across the cortex [30], [32]: Two major significant manganese-enhanced points in the profile were located in layer II and in the transition between IV and V. Finally, the SIRs in each layer were averaged, and these averages were fed into the statistical analyses. The same procedure was repeated for Sens and Vis. Based on percentage depth of cortical layers in previous cytoarchitectural studies [33], [34], each layer depth was determined to be consistent with the average signal intensity profile from MEMRI across the cortex [30], [32]. For Sens, layer I was defined between 0 µm to 175 µm, layer II/III was between 175 µm to 575 µm, layer IV was between 575 µm to 850 µm, layer V was between 850 µm to 1450 µm, and layer VI was between 1450 µm to 1900 µm. For Vis, layer I was defined between 0 µm to 150 µm, layer II/III was between 150 µm to 575 µm, layer IV was between 575 µm to 800 µm, layer V was between 800 µm to 1125 µm, and layer VI was between 1125 µm to 1500 µm.

### 2.6. Statistical analyses

Statistical tests for the mean SIRs of the cortical layers were carried as follows. As some parameters did not adhere to normal distribution, comparison of variables was performed using Mann-Whitney *U*-tests, and the relationships between variables were calculated using Spearman's nonparametric correlations. Firstly, to test for the effects of acoustic stimulation in the layers of the auditory cortex, we used general linear models (GLM in the Statistical Package for the Social Sciences 20.0, Chicago, IL, USA) in which the dependent measures were the mean SIRs for 5 cortical layers (I, II/III, IV, V, and VI) in the left and right hemispheres. Within-subject factors were cortical layers (5 levels) and hemispheres (2 levels). Adjustments of degrees of freedom were employed according to Greenhouse-Geisser where appropriate. Secondly, the *post hoc* Mann-Whitney *U*-tests for the left and right Aud layers were performed to compare the mean SIRs between stimulated and unstimulated rats. We repeated these analyses in the layers of the Sens and Vis. Thirdly, the associations between the mean SIRs of the left and right layers in the cortex were analyzed using Spearman's rank correlations, which yielded correlation map for each cortex. The correlation map was made based on the correlation coefficients which are obtained in the scatter plot of mean SIRs between two Aud layers; the higher linearity in the scatter plot, the closer color to red in the correlation map (Figure S3).

Finally, to compare the correlation coefficients of the mean SIRs in left and right layers between the condition with (STIM) and without (NOSTIM) auditory stimulation, we used Mann-Whitney *U*-tests. These analyses were conducted to compare the correlation strengths of interhemispheric associations between STIM and NOSTIM.

## Results

### 3.1. Comparisons of manganese uptake between STIM and NOSTIM

#### 3.1.1. Stimulus-dependent activities in auditory pathway

The mean SIRs of ROIs in the auditory pathway were compared between STIM and NOSTIM. As shown in Table 1, the SIRs for STIM were significantly higher than those for NOSTIM (*P*<0.05) in the ROIs including bilateral MGB, and left CN, left superior olive (SO), left lateral lemniscus (LL), and right IC. As a negative control, we also compared the mean SIRs of ROIs in olfactory pathway such as OB and Pir/LOT, which showed no significant differences between STIM and NOSTIM.

**Table 1:** Comparison of SIRs between STIM and NOSTIM in the brain regions of auditory and olfactory pathways.

	STIM (*n* = 16)	NOSTIM (*n* = 16)	*z*		*P*
	Mean (SD)	Mean (SD)			
Left					
CN	1.41 (0.18)	1.17 (0.16)		3.22	0.001*
LL	1.18 (0.16)	1.05 (0.11)		2.28	0.02*
SO	1.25 (0.17)	1.10 (0.10)		2.77	0.006*
IC	1.25 (0.20)	1.17 (0.17)		1.26	0.2
MGB	1.20 (0.19)	1.03 (0.12)		2.54	0.01*
OB	2.66 (0.78)	2.93 (0.49)	–	1.68	0.09
Pir/LOT	1.44 (0.26)	1.37 (0.21)		1.68	0.09
Right					
CN	1.38 (0.28)	1.23 (0.16)		1.83	0.07
LL	1.10 (0.18)	1.06 (0.13)		0.62	0.5
SO	1.11 (0.22)	1.11 (0.17)		0.21	0.8
IC	1.29 (0.21)	1.11 (0.15)		2.51	0.01*
MGB	1.34 (0.19)	1.03 (0.15)		3.71	<0.001*
OB	2.76 (0.60)	2.87 (0.50)	–	0.81	0.4
Pir/LOT	1.40 (0.22)	1.36 (0.15)		0.21	0.8

Mann-Whitney *U*-tests were used to compare the mean SIRs of brain regions. The *z*-values were calculated from Mann-Whitney's *U*-values and their standard deviations. SIR is the normalized signal intensity of each ROI to its adjacent Temporalis muscles.

*P<0.05.

#### 3.1.2. Stimulus-dependent activities in cortical layers

The primary auditory, sensory, and visual cortices in the left and right hemispheres were divided into 5 layers (I, II/III, IV, V, and VI) after linearization of the cortices. The mean SIRs for NOSTIM and STIM in each layer (5) and hemisphere (2) were compared using multivariate GLM analyses, which were conducted for auditory, sensory, and visual cortex, separately (Table S1). Adjustments of degrees of freedom were employed according to Greenhouse-Geisser where appropriate.

In the primary auditory cortex, there was a significant interaction of layer×stimulation on the mean SIRs, which suggests that stimulation had a layer-specific effect on the mean SIR. As for the primary sensory and visual cortices, the GLM analyses showed significant effects of hemisphere×layer×stimulation. Based on the significant effects of the layer×stimulation in the mean SIRs in Aud, we conducted *post hoc* Mann-Whitney *U*-tests to compare the mean SIR between STIM and NOSTIM in each layer. We also conducted *post hoc* Mann-Whitney *U*-tests over the mean SIRs of left and right layers in Sens and Aud based on the significant hemisphere×layer×stimulation interactions.

**Figure 1:**
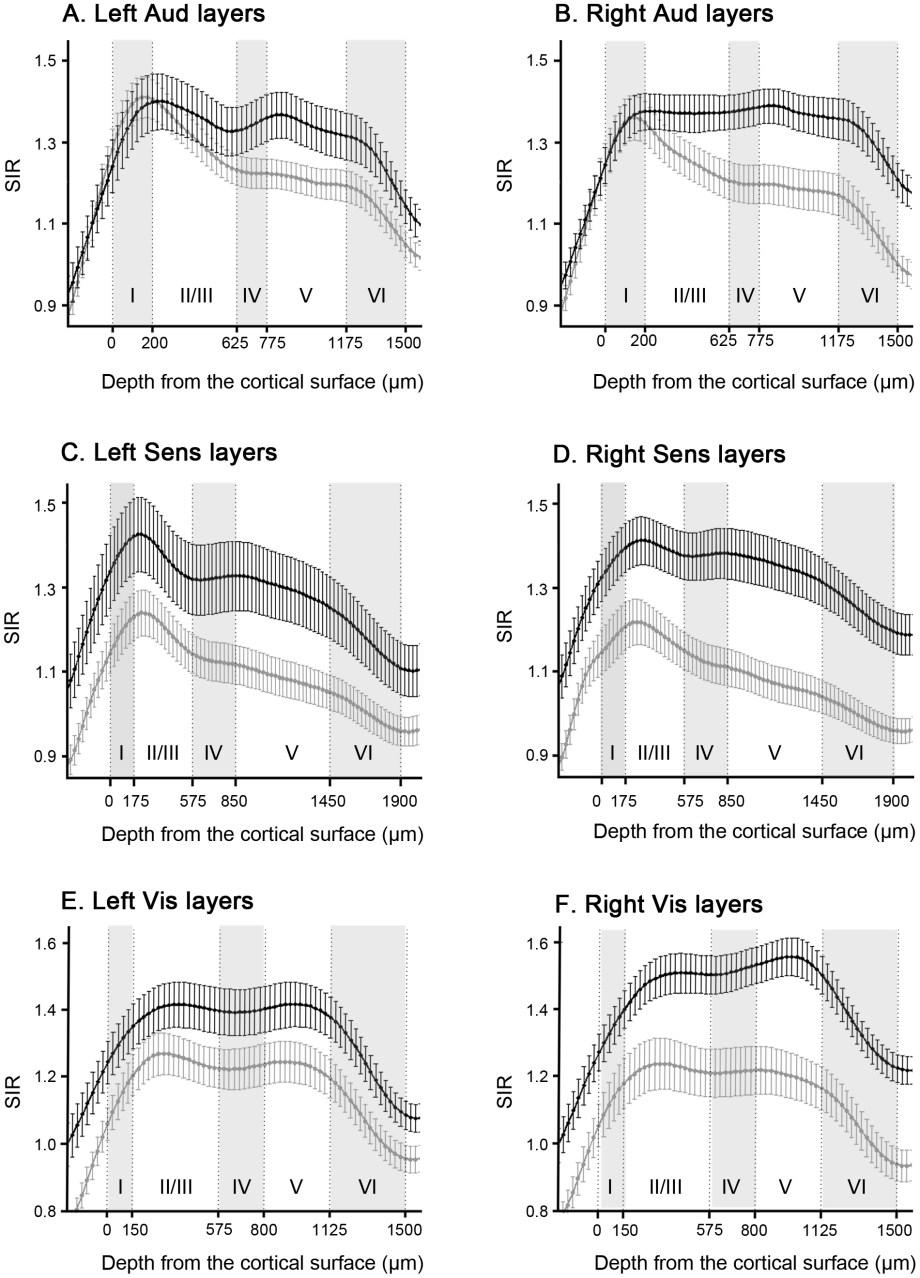
Profiles of signal intensity ratio (SIR) as a function of depth from the surface of cortices. (A) Left Aud layers (B) Right Aud layers (C) Left Sens layers (D) Right Sens layers (E) Left Vis layers (F) Right Vis layers. Black lines and bars indicate the SIRs of the rats with acoustic stimulation; gray lines and bars indicate those without stimulation. Error bars represent the standard error of the mean.

The results of *post hoc* Mann-Whitney *U*-tests are shown in Table 2, 3 and 4. In the Aud (Table 2), we observed significant differences of the mean SIRs in right layer IV, V, and VI between STIM and NOSTIM, and the left Aud layer V and VI also showed marginally significant difference (see also Figure 1A and 1B). In the Sens (Table 3), all the left and right layers showed significant differences in the mean SIRs (see also Figure 1C and 1D). In the Vis (Table 4), the mean SIRs in the left layer I, V, and VI and all the right layers showed significant difference, and left layer IV also showed marginally significant difference (see also Figure 1E and 1F).

**Table 2:** Layer-specific comparison of SIRs between STIM and NOSTIM in the primary auditory cor*t*ex.

	STIM (*n* = 16)	NOSTIM (*n* = 16)	*z*		*P*
	Mean (SD)	Mean (SD)			
Left Aud layers					
I	1.33 (0.28)	1.37 (0.20)	–	0.58	0.6
II/III	1.37 (0.26)	1.32 (0.16)		0.55	0.6
IV	1.34 (0.23)	1.23 (0.15)		1.53	0.1
V	1.35 (0.23)	1.21 (0.14)		1.90	0.06
VI	1.26 (0.20)	1.14 (0.14)		1.90	0.06
Right Aud layers					
I	1.33 (0.18)	1.33 (0.22)	–	0.09	0.9
II/III	1.38 (0.17)	1.27 (0.18)		1.45	0.15
IV	1.38 (0.17)	1.20 (0.18)		2.54	0.01*
V	1.38 (0.17)	1.19 (0.19)		2.51	0.01*
VI	1.31 (0.19)	1.11 (0.17)		2.69	0.007*

Mann-Whitney *U*-tests were used to compare the mean SIRs of Aud layers. The *z*-values were calculated from Mann-Whitney's *U*-values and their standard deviations. SIR is the normalized signal intensity of each ROI to its adjacent Temporalis muscles.

*P<0.05.

**Table 3:** Layer-specific comparison of SIRs between STIM and NOSTIM in the primary sensory cortex.

	STIM (*n* = 16)	NOSTIM (*n* = 16)	*z*	*P*
	Mean (SD)	Mean (SD)		
Left Sens layers				
I	1.39 (0.35)	1.20 (0.21)	2.13	0.03*
II/III	1.38 (0.34)	1.20 (0.20)	2.17	0.03*
IV	1.33 (0.33)	1.13 (0.18)	2.20	0.03*
V	1.30 (0.31)	1.09 (0.17)	2.36	0.02*
VI	1.18 (0.27)	1.01 (0.15)	2.28	0.02*
Right Sens layers				
I	1.36 (0.23)	1.18 (0.22)	2.17	0.03*
II/III	1.40 (0.22)	1.19 (0.19)	2.47	0.01*
IV	1.38 (0.23)	1.13 (0.17)	2.96	0.003*
V	1.35 (0.24)	1.08 (0.17)	3.11	0.002*
VI	1.25 (0.21)	1.00 (0.13)	3.22	0.001*

Mann-Whitney *U*-tests were used to compare the mean SIRs of Sens layers. The *z*-values were calculated from Mann-Whitney's *U*-values and their standard deviations. SIR is the normalized signal intensity of each ROI to its adjacent Temporalis muscles.

*P<0.05.

**Table 4:** Layer-specific comparison of SIRs between STIM and NOSTIM in the primary visual cortex.

	STIM (*n* = 16)	NOSTIM (*n* = 16)	*z*	*P*
	Mean (SD)	Mean (SD)		
Left Vis layers				
I	1.31 (0.26)	1.14 (0.22)	1.98	0.05*
II/III	1.41 (0.27)	1.25 (0.24)	1.49	0.14
IV	1.40 (0.27)	1.23 (0.24)	1.90	0.06
V	1.41 (0.26)	1.24 (0.24)	2.36	0.02*
VI	1.22 (0.20)	1.06 (0.19)	2.39	0.02*
Right Vis layers				
I	1.34 (0.22)	1.13 (0.28)	2.43	0.02*
II/III	1.50 (0.23)	1.23 (0.30)	2.81	0.005*
IV	1.52 (0.22)	1.22 (0.29)	3.15	0.002*
V	1.55 (0.22)	1.20 (0.27)	3.41	<0.001*
VI	1.34 (0.18)	1.05 (0.23)	3.41	<0.001*

Mann-Whitney *U*-tests were used to compare the mean SIRs of Vis layers. The *z*-values were calculated from Mann-Whitney's *U*-values and their standard deviations. SIR is the normalized signal intensity of each ROI to its adjacent Temporalis muscles.

*P<0.05.

### 3.2. Comparison of manganese uptake correlations among cortical layers between STIM and NOSTIM

**Figure 2:**
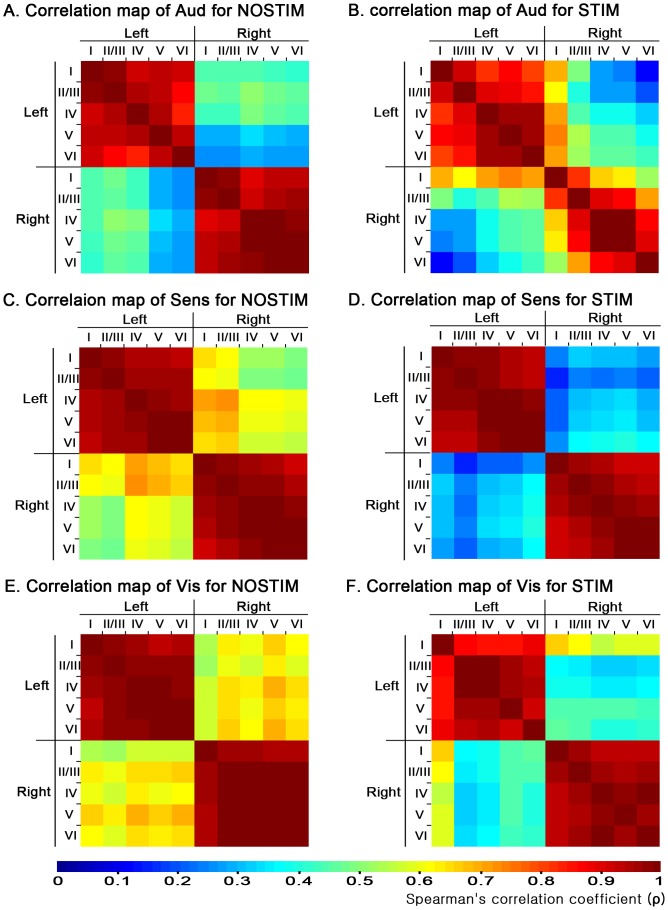
Spearman's rank correlation maps between the mean SIRs of each layer in cortices. (A) Correlation map of Aud for NOSTIM (B) Correlation map of Aud for STIM (C) Correlation map of Sens for NOSTIM (D) Correlation map of Sens for STIM (E) Correlation map of Vis for NOSTIM (F) Correlation map of Vis for STIM. The correlation coefficient, ρ, is indicated with color maps ranging from blue to red; blue and red indicates the weakest and strongest correlation, respectively. A strong correlation between two layers indicates that the manganese accumulations in the two are likely to be linearly proportional to each other.

The stimulus-dependent activities in various cortices were observed with responding to acoustic stimulation, and the activities were different across layers and hemispheres. To investigate association across layers and interhemispheric association of each cortex, we performed correlation analysis of manganese uptake for Aud, Sens, and Vis separately, and compared the associations between STIM and NOSTIM (Figure 2). A mean SIR value in a brain region is directly related to the regional manganese accumulation. Hence, correlation analysis of mean SIR values between two brain regions reflects the strength of manganese-uptake association between the two regions. For example, a highly positive correlation between right Aud layer V and VI for STIM is shown in red in the correlation map and a strong linear relationship in the scatter plot of Figure S3. This means that it is highly probable for manganese to accumulate in the layer VI when it is accumulated in the layer V.

In comparison between STIM (Figure 2B, 2D, and 2F) and NOSTIM (Figure 2A, 2C, and 2E), all the Aud, Sens, and Vis layers showed high coefficients of ipsilateral correlations (left-left and right-right) regardless of the acoustic stimulation (all ρ's>0.81) with one exception of the correlation between right Aud layer I and VI for STIM. On the other hand, contralateral correlations (left-right) among each Aud, Sens, and Vis layers showed different patterns between STIM and NOSTIM. The contralateral correlations of Aud layers were slightly but not significantly stronger for STIM than for NOSTIM (STIM, 0.44±0.17 [mean ± SD of correlation coefficients, ρ], NOSTIM, 0.39±0.09, *P* = 0.38, Mann-Whitney *U*-tests), which is also noticed by the difference of colors in correlation map between Figure 2A and 2B. However, the Sens and Vis layers showed significantly lower contralateral correlation coefficients for STIM than for NOSTIM (Sens, STIM, 0.60±0.07, Sens, NOSTIM, 0.29±0.06, *P*<0.001; Vis, STIM, 0.62±0.05, Vis, NOSTIM, 0.43±0.10, *P*<0.001).

## Discussion

### 4.1. Response of auditory-related brain regions to acoustic stimulation

The current study demonstrates the enhancements of SIRs in Aud of rats after auditory stimulation. Only deep layers, such as the right layer IV, V, and VI, showed greater SIRs in STIM rats than in NOSTIM rats (Figure 1 and Table 2). In addition, our results indicate that manganese accumulation was greater with stimulation in auditory pathways including CN, SO, LL, IC, and MGB (Table 1). In previous MEMRI studies, the auditory pathways were investigated by direct cochlear injection without acoustic stimulation [14] or by functional mapping with acoustic stimulation [15], [16], [20]. However, these pathways included only the brainstem and diencephalon regions. An enhanced accumulation of manganese ions in the Aud with auditory stimulation has not yet been observed. Using cortex linearization [30], we extracted the mean SIRs for each layer, and our study found enhanced activity in the deep Aud layers (IV, V, and V) as well as auditory pathways (CN, SO, LL, IC, and MGB) under acoustic stimulation. In the previous neural tracing studies, the deep layers of rodent auditory cortex were known to have intensive neural connections with auditory pathways, which was revealed by reciprocal projections of the layers IV and VI with the MGB [2], [10], [35]–[37] and corticocollicular projections from layers V and VI to the IC [1], [38]. These results in the previous studies are consistent with our findings.

### 4.2. Various cortical responses to auditory stimulation

The present study showed the response of various cortical regions to acoustic stimulation, including Sens and Vis as well as Aud. As shown in Figure 1 and Table 3 and 4, the mean SIRs in the all the left and right layers of Sens were significantly higher for STIM than for NOSTIM and, in the case of Vis, all layers except for left layer II/III and IV had greater mean SIRs for STIM than for NOSTIM. Previous neural tracing study revealed the neuronal connection of Aud with Sens and Vis [3]. Also, previous electrophysiology studies demonstrated that Vis responds to auditory stimulation [5], [6], and showed that auditory and sensory system are deeply related [7]. These results in neural tracing and electrophysiology studies suggest that some regions of the primary sensory and visual cortices can be activated by acoustic stimulation, which supports our findings even though the biological basis of increased SIRs in Sens and Vis for STIM needs further investigation.

Based on various cortical activities under auditory stimulation, we performed a correlation analysis of manganese uptake between cortical layers within each Aud, Sens, and Vis. By comparing the correlation patterns of STIM with those of NOSTIM, we investigated how cortical associations in each Aud, Sens, and Vis were different between with and without auditory stimulation. With the correlation analysis, the contralateral correlation of Aud showed a slight but not significant increase with acoustic stimulation, whereas Sens and Vis showed significantly weaker contralateral correlations (Figure 2). This suggests that Sens and Vis layers establish weaker associations with their contralateral counterparts in response to acoustic stimulation. It supports the possibility that the Aud, Sens, and Vis have different associations for auditory processing in the brain network.

### 4.3. Limitations

There were some limitations to correlation analysis. We conducted correlation analysis between the manganese accumulations in the Aud, Sens, and Vis layers. The manganese accumulation is a combined result of several sources including neural activity and BBB permeability [17], and these sources could not be clearly distinguished in our study. Hence, the exact cause for manganese accumulation needs further investigation. However, it is reasonable to speculate that the increased manganese accumulation measured by the SIR with the stimulation is most likely due to neural activity. On the other hand, in the absence of acoustic stimulation, BBB permeability can be considered as the dominant factor in manganese accumulation. Even though this assumption regarding the cause of manganese accumulation for STIM and NOSTIM is acceptable, the results should be interpreted with caution.

## Conclusions

Our MEMRI study, with the aid of cortex linearization and layer analysis, demonstrated cortical responses of Aud, Sens, and Vis layers to acoustic stimulation in two ways. The first was the enhanced manganese accumulation after acoustic stimulation in each Aud, Sens, and Vis. The second was the comparison of cortical network between STIM and NOSTIM. Vis and Sens had weaker contralateral correlations for STIM. Based on these results, our study demonstrates that the primary auditory, sensory, and visual cortices show enhanced activity to the auditory stimulation, and suggests that these cortices have different associations for auditory processing in the brain network.

## Supporting Information

Figure S1 **Cortex linearization.** (A) On the coronal view, the Aud was delineated to the atlas [28]. Cyan points were located at intervals of 1 mm for reference coordinates. (B) For flattening the cortex, the outline of the cortex (red) was fitted to a fourth order polynomial, from which the perpendicular lines (yellow) were drawn. (C) The flattened cortex. In (B) and (C), the green and blue lines were located at the same points, and red fitting line in (B) was linearized in (C). (D) The cortical layers of Aud. Layer I was presented in yellow lines; Layer II/III, light purple; Layer IV, blue; Layer V, pink; Layer VI, green. Br indicates Bregma.
(TIF)

Figure S2 **Temporalis Muscle (TM) ROIs used for normalizing the mean signal intensity (SI) of each adjacent ROIs.** (A) TM for Aud (B) TM for Sens (C) TM for Vis (D) TM for OB (E) TM for Pir/LOT (F) TM for MGB (G) TM for IC (H) TM for CN, SO, and LL. Red, cyan, yellow, and blue indicates left adjacent TM ROI, right adjacent TM ROI, left ROI, and right ROI, respectively.
(TIF)

Figure S3 **Correlation map generation.** (A) Correlation map of Aud for STIM (B) Scatter plot of mean SIRs between two Aud layers. The correlation map was made based on the Spearman's rank correlation coefficients obtained in the scatter plot of mean SIRs between two Aud layers; the higher linearity in the scatter plot, the closer color to red in the correlation map. Hence, the color in the correlation map indicates the strength of correlation; red and blue corresponds to a strong and weak correlation of manganese uptake, respectively.
(TIF)

Table S1 **Comparison of the mean SIRs for NOSTIM and STIM in each layer and hemisphere of Aud, Sens and Vis.** The mean SIRs for NOSTIM and STIM in each layer (5 levels) and hemisphere (2 levels) were compared using multivariate GLM analyses. SIR is the normalized signal intensity of each ROI to its adjacent Temporalis muscles. *P<0.05.
(DOC)
