# Supplementary figures and images for: Response of the Primary Auditory and Non-Auditory Cortices to Acoustic Stimulation: A Manganese-Enhanced MRI Study
Source: PLoS One. 2014 Mar 11;9(3):e90427. doi: 10.1371/journal.pone.0090427 (PMC3949704; doi:10.1371/journal.pone.0090427)

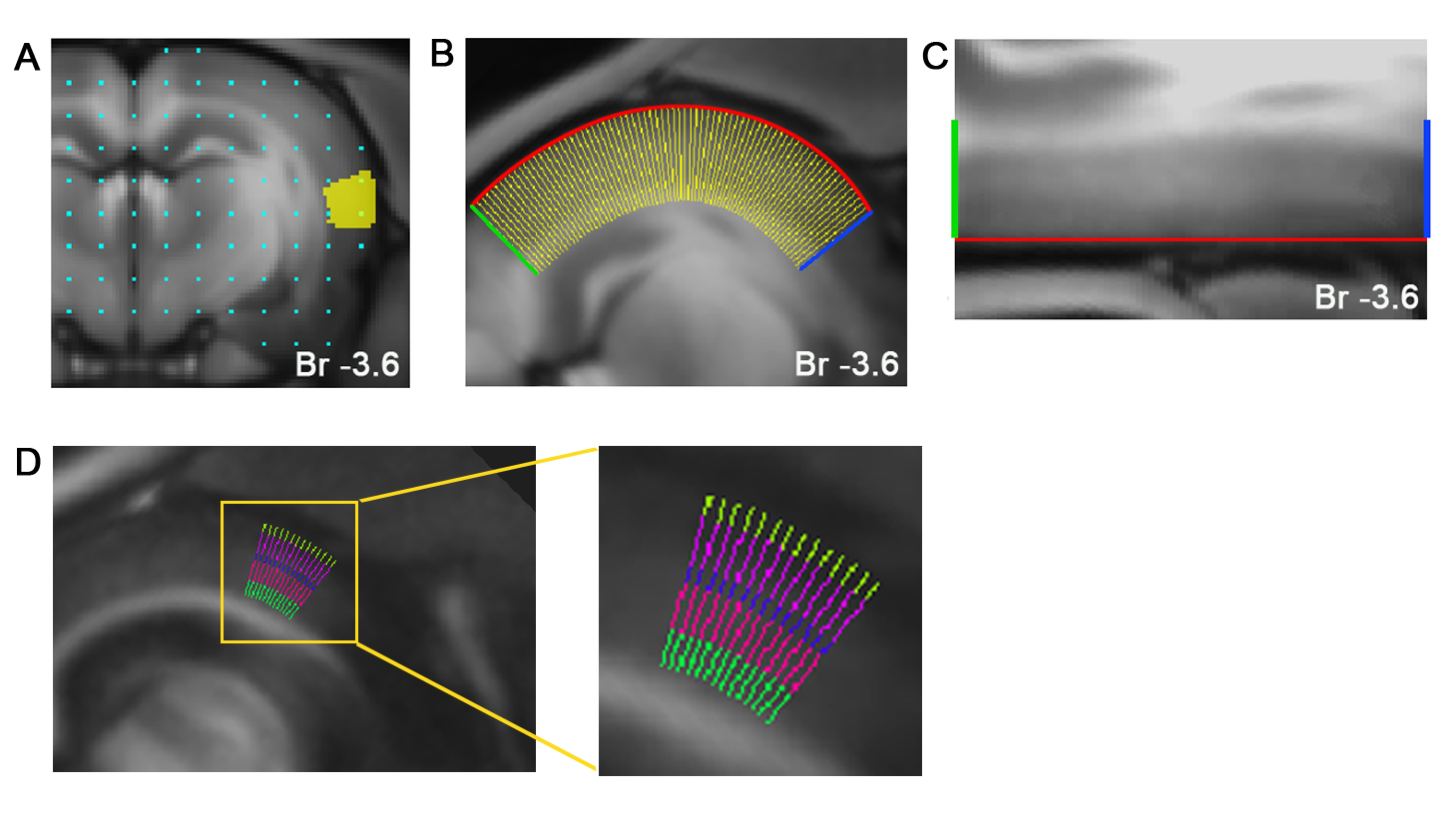

Supplement: Figure S1 — Cortex linearization. (A) On the coronal view, the Aud was delineated to the atlas [28]. Cyan points were located at intervals of 1 mm for reference coordinates. (B) For flattening the cortex, the outline of the cortex (red) was fitted to a fourth order polynomial, from which the perpendicular lines (yellow) were drawn. (C) The flattened cortex. In (B) and (C), the green and blue lines were located at the same points, and red fitting line in (B) was linearized in (C). (D) The cortical layers of Aud. Layer I was presented in yellow lines; Layer II/III, light purple; Layer IV, blue; Layer V, pink; Layer VI, green. Br indicates Bregma. (TIF) [file pone.0090427.s001.tif]

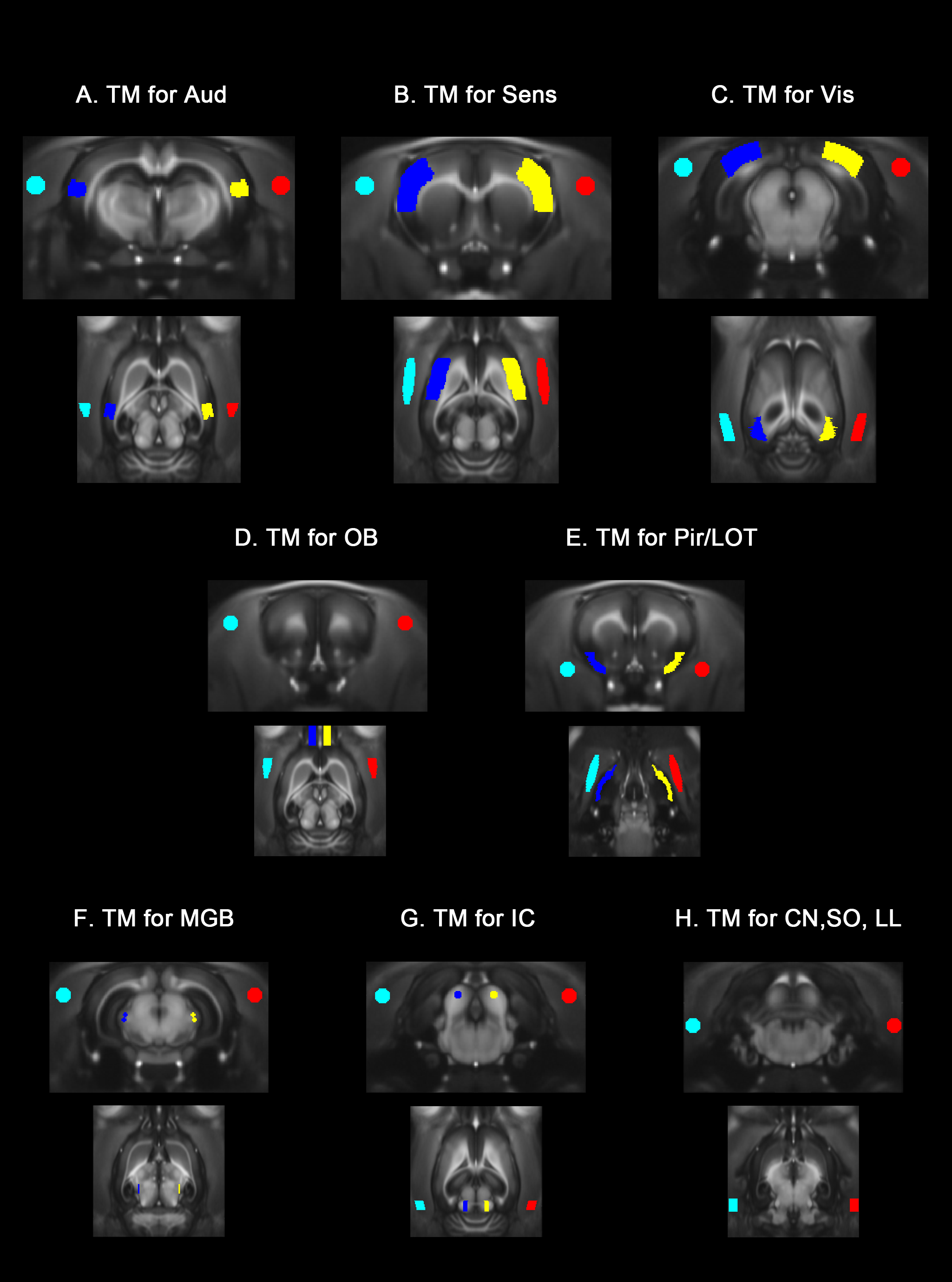

Supplement: Figure S2 — Temporalis Muscle (TM) ROIs used for normalizing the mean signal intensity (SI) of each adjacent ROIs. (A) TM for Aud (B) TM for Sens (C) TM for Vis (D) TM for OB (E) TM for Pir/LOT (F) TM for MGB (G) TM for IC (H) TM for CN, SO, and LL. Red, cyan, yellow, and blue indicates left adjacent TM ROI, right adjacent TM ROI, left ROI, and right ROI, respectively. (TIF) [file pone.0090427.s002.tif]

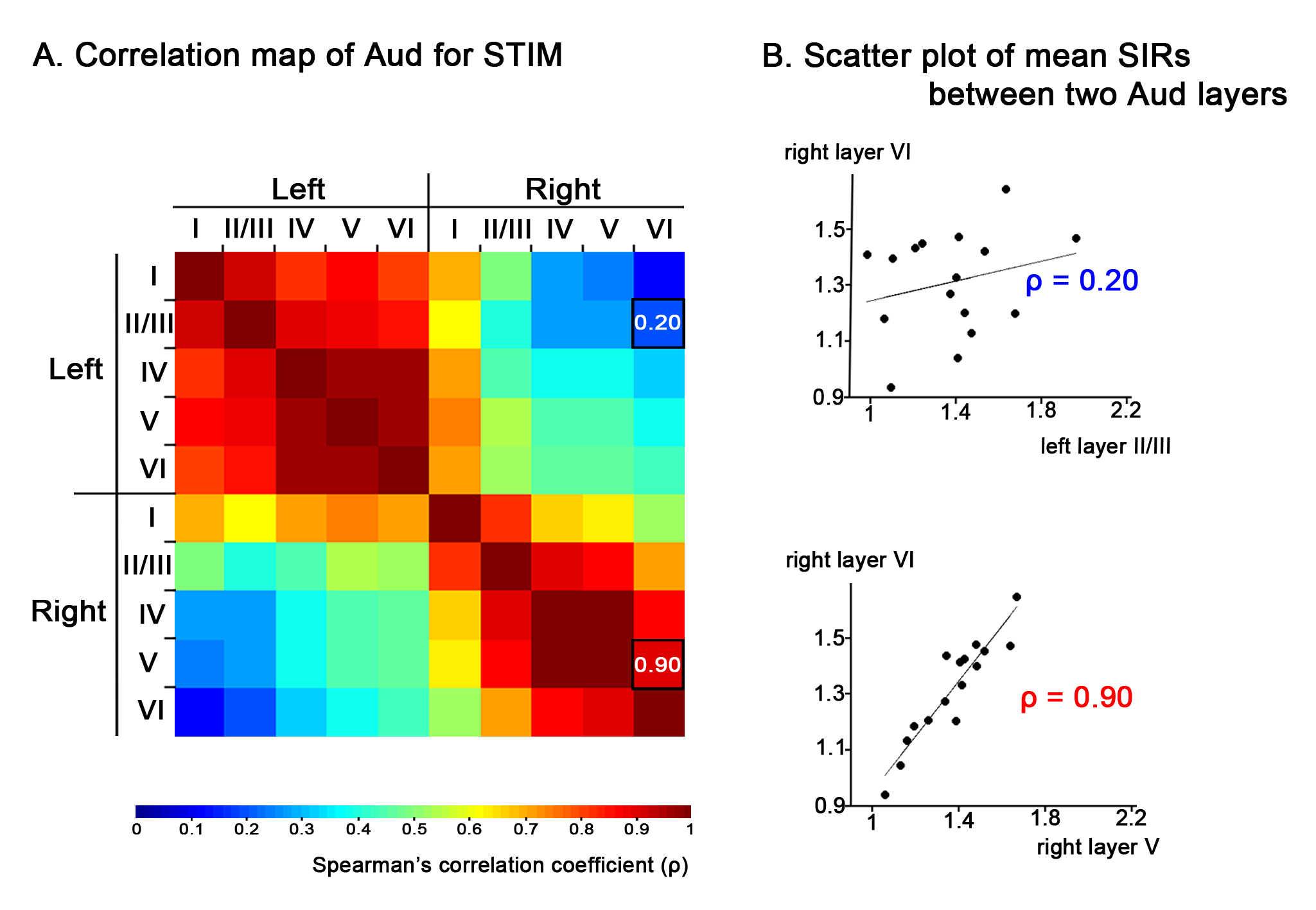

Supplement: Figure S3 — Correlation map generation. (A) Correlation map of Aud for STIM (B) Scatter plot of mean SIRs between two Aud layers. The correlation map was made based on the Spearman's rank correlation coefficients obtained in the scatter plot of mean SIRs between two Aud layers; the higher linearity in the scatter plot, the closer color to red in the correlation map. Hence, the color in the correlation map indicates the strength of correlation; red and blue corresponds to a strong and weak correlation of manganese uptake, respectively. (TIF) [file pone.0090427.s003.tif]
