# Supplementary material for: Response of the Primary Auditory and Non-Auditory Cortices to Acoustic Stimulation: A Manganese-Enhanced MRI Study
Source: PLoS One. 2014 Mar 11;9(3):e90427. doi: 10.1371/journal.pone.0090427 (PMC3949704; doi:10.1371/journal.pone.0090427)
Supplement: Table S1 — Comparison of the mean SIRs for NOSTIM and STIM in each layer and hemisphere of Aud, Sens and Vis. The mean SIRs for NOSTIM and STIM in each layer (5 levels) and hemisphere (2 levels) were compared using multivariate GLM analyses. SIR is the normalized signal intensity of each ROI to its adjacent Temporalis muscles. *P<0.05. (DOC) [file pone.0090427.s004.doc]

**Table S1.** Comparison of the mean SIRs for NOSTIM and STIM in each layer and hemisphere of Aud, Sens and Vis.

|  | Aud | | Sens | | Vis | |
| --- | --- | --- | --- | --- | --- | --- |
|  | *F* | *P* | *F* | *P* | *F* | *P* |
| Stimulation | 3.37 | 0.08 | 9.40 | 0.005* | 8.21 | 0.008* |
| Hemisphere | 0.02 | 0.90 | 0.09 | 0.77 | 1.56 | 0.22 |
| Hemisphere × Stimulation | 0.81 | 0.38 | 0.27 | 0.60 | 3.22 | 0.08 |
| Layer | 32.69 | <0.001* | 93.32 | <0.001* | 136.26 | <0.001* |
| Layer × Stimulation | 17.08 | <0.001* | 2.45 | 0.12 | 3.22 | 0.04* |
| Hemisphere × Layer | 3.11 | 0.04* | 11.09 | <0.001* | 6.24 | 0.001* |
| Hemisphere × Layer × Stimulation | 0.99 | 0.39 | 7.89 | 0.001* | 8.80 | <0.001* |

The mean SIRs for NOSTIM and STIM in each layer (5 levels) and hemisphere (2 levels) were compared using multivariate GLM analyses. SIR is the normalized signal intensity of each ROI to its adjacent Temporalis muscles. **P <* 0.05.
